# Wandering permanent pacemaker generators in children: a case series

**DOI:** 10.1186/1752-1947-2-163

**Published:** 2008-05-18

**Authors:** Hilal Al Sabti, Raj Gopal Menon, Madan Mohan Maddali, John Valliattu

**Affiliations:** 1 Department of Cardiothoracic Surgery, Sultan Qaboos University Hospital, Muscat, Sultanate of Oman; 2 Department of Cardiothoracic Surgery, Royal Hospital, Muscat, Sultanate of Oman; 3 Department of Anesthesia, Royal Hospital, Muscat, Sultanate of Oman; 4 Department of Cardiothoracic Surgery, Royal Hospital, Muscat, Sultanate of Oman

## Abstract

**Introduction:**

Epicardial permanent pacemaker generators are implanted some times in the abdominal wall in pediatric age groups.

**Case presentation:**

Three permanent epicardial pacemakers that migrated in an unusual manner producing intraabdominal complications are reported.

**Conclusion:**

The different clinical presentations of pacemaker migration in the pediatric age groups are highlighted and a few suggestions are made for avoiding such a complication.

## Introduction

Cardiac pacing in the pediatric population typically results from bradycardia produced by sinus node dysfunction or atrioventricular [AV] block [1]. Although endocardial pacing requires less extensive surgery than does epicardial lead implantation, there is concern about vascular obstruction, AV valve integrity, and the limitations of lead accommodation in relation to somatic growth especially in early childhood. Hence permanent epicardial pacing is often chosen in early age groups. We report on three cases of migrating pacemakers each of which presented in a very unusual manner. The course of events, their individual management and preventive aspects are described.

## Case presentation 1

A 6-year-old boy was investigated for intermittent colicky pain in his abdomen. The child was afebrile with no signs of intestinal obstruction.

During infancy, the child had undergone repair of an interrupted aortic arch and ventricular septal defect [VSD] closure as two staged procedures. Following the second operation, the child developed a complete heart block for which an adult epicardial pacemaker system (Sigma SSR 203, Medtronic, Inc. Minneapolis, USA) was implanted with the pulse generator in the epigastric region.

**Figure 1:**
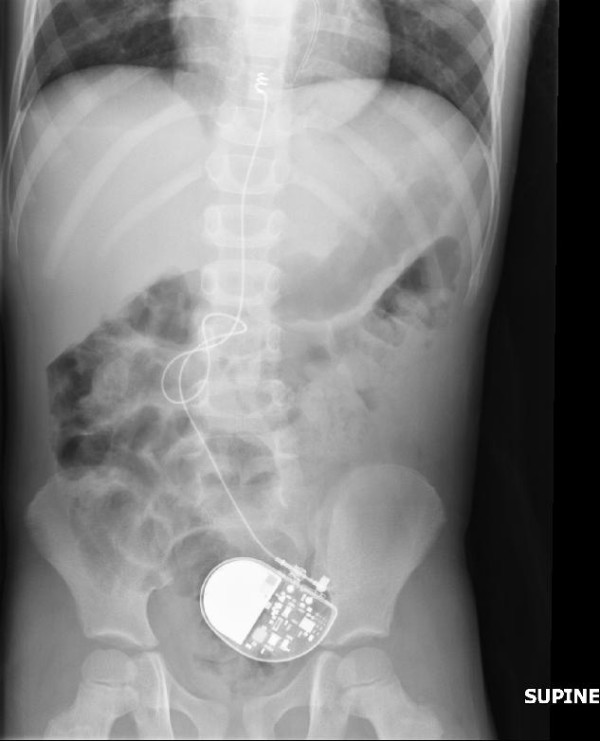
X-ray of the abdomen illustrating the migrated pacemaker generator in the pelvis.

When the child presented with abdominal manifestations it was found that the epigastric pocket where the generator was placed was empty. An abdominal X-ray (Figure 1) showed the pacemaker lying in the pelvis confirming the diagnosis of intraperitoneal pacemaker migration.

**Figure 2:**
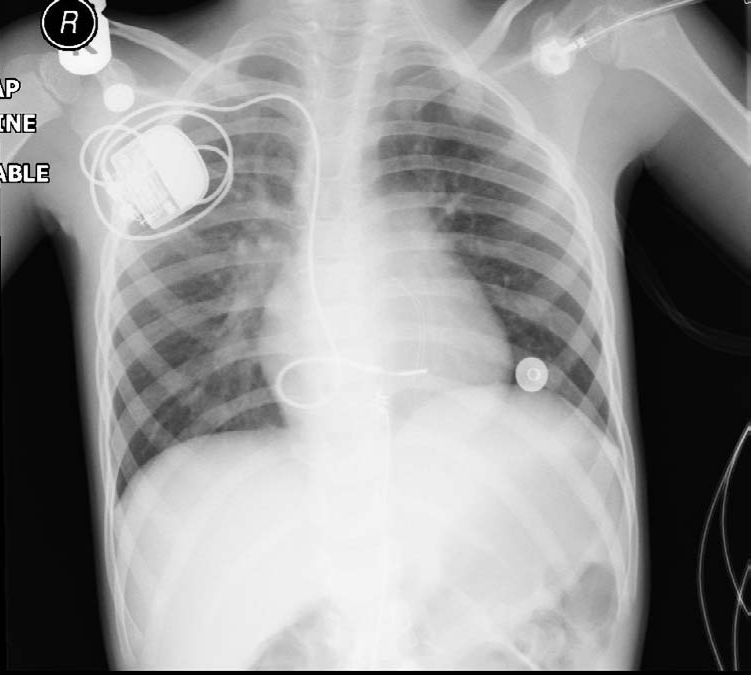
X-ray depicting the endocardial pacemaker inserted through the right subclavian vein.

Under general anesthesia, endovenous endocardial electrodes were inserted and a subpectoral generator placement was performed (Figure 2). At the same time, the old pulse generator was retrieved through a laparotomy. Resection and anastamosis of the small bowel had to be performed to release the bowel from the adherent and entangled pacing wires. The child had a smooth recovery and was discharged home on the sixth postoperative day.

## Case presentation 2

A seven-year-old girl presented with colicky abdominal pain of six days duration with no signs of localization or intestinal obstruction. She was afebrile with a normal white cell count. An electrocardiogram showed AV dissociation with a ventricular rate of 60 beats per minute. There were pacing spikes of 7 mm on the electrocardiogram ECG] with no relation to the QRS complexes. An antero-posterior abdominal X-ray showed a metallic shadow in the pelvic cavity and chest X-ray revealed fractured leads close to the heart leading to a diagnosis of a migrated pacemaker.

This child was delivered at 32 weeks of gestational age by an emergency cesarean section. Electrocardiogram showed a congenital complete AV block with a ventricular rate of 60 per minute. In the immediate neonatal period, patent ductus arteriosus [PDA], which was detected by transthoracic echocardiography, was ligated and an epicardial pacemaker system [Prodigy, SR 8160, VVI, Medtronic, Inc, Minneapolis, USA] with a pulse rate of 100 per minute was implanted. After 5 years the battery expired and the pacemaker was replaced with an adult sized rate responsive VVI pacemaker [Sigma SSR 203, Medtronic, Inc. Minneapolis, USA] that was set to discharge at a pulse rate of 100 per minute. This was implanted in the epigastric space behind the rectus abdominis muscle.

Under general anesthesia, a left anterolateral thoracotomy was done and a new VVI pacemaker [Microny II SR + Model 2525T St Jude Medical, Inc. Sylmar, CA, USA] with epicardial leads, programmed to deliver 100 beats per minute, was implanted in the left infraclavicular area superficial to the pectoralis major muscle. The migrated pacemaker and its fractured wires were retrieved by laparotomy. The postoperative course was smooth and child was discharged five days later.

## Case presentation 3

A 2-year-old girl presented with complaints of diarrhea, vague abdominal pain, low-grade fever of one-week duration and history of passage of a foreign body per rectum. When this child was 8 months old, a permanent bipolar cardiac pacemaker [Prodigy, VVI, Medtronic, Inc, Minneapolis, USA] was inserted in an epigastric pocket below the rectus abdominis as she had developed complete heart block following repair of an atrioventricular septal defect.

In this child, the pacemaker generator was found extruded through the rectum but hanging by the pacing wires and was functional. There were no clinical or radiological signs of bowel perforation. Under antibiotic cover, a new epicardial pacemaker system was inserted through a left anterolateral thoracotomy and the pacemaker generator was hitched to the rib with non absorbable sutures to prevent migration. The old pacing wires were cut and were pulled out from the rectum. The postoperative course was uneventful and the child was discharged two days later.

## Discussion

Permanent pacemaker implantation in children is associated with technical problems related to the size of the patient and the pacemaker, the child's growth, and the need for repeated pacemaker replacements due to the short battery life.

In early infancy, a single chamber pacemaker is preferred due to its relative smaller size as compared to dual chamber pacemakers. Single chamber pacemakers have longer battery duration as well. However, the selection of pacemaker depends on its effect on the child's cardiac output. These pulse generators are implanted in the abdominal wall in the epigastric region due to the disproportionate size of the pulse generators and for easy access if the need for replacement occurs following battery depletion.

Since children have little subcutaneous tissue, they can easily palpate the pacemaker and tend to play with it, causing breakage or displacement of pacing wires resulting in pacemaker dysfunction [2]. Continuous traction from twiddling the pacemaker can result in displacement or twisting of the pacemaker leads [3] leading to the migration of the pacemaker generators into neighboring body cavities.

A pacemaker that has migrated into the abdomen can cause partial or complete intestinal obstruction [4] might cause colonic perforation [5] or get extruded [6].

Despite the fact that differences between pediatric and adult pacing have narrowed, implantation procedures in children require special considerations. Patients are physically smaller and they often suffer from complex cardiac defects. Selection of appropriate pacemaker systems is important in children, as there are no pacemakers but only some electrodes designed specially for pediatric pacing.

What makes the pacemakers in children prone to complications like migration or displacement? Firstly, in children, the subcutaneous tissue can be an important factor, emphasizing the importance of adapting the subcutaneous pocket to the exact size of the pulse generator. Fixation of the pulse generator with a ligature during the implantation would probably prevent the generator from being rotated by the patient but it is difficult to anchor the pacemaker in the abdominal wall or axilla due to lax surrounding tissues. A pulse generator fixed too close to the clavicle can result in pain and discomfort. Many believe that a tightly fitting pocket without redundant space around the generator would provide adequate fixation in the majority of patients. However, fixation of the pulse generator can be considered in children especially with mental disorders, confusion or very lax subcutaneous tissues. The second reason that predisposes to migration of pacemakers in children is the relative bulk of the pacemaker leading to a tendency to slip by gravity from their original placement site and migrate.

Any child with a malfunctioning pacemaker must be investigated systematically. Usually symptoms of low cardiac output are present. The one important sign suggesting the possibility of migration is an inability to palpate the pacemaker at the site of placement i.e. an 'empty' pocket. A 12 lead ECG can help to show failure to capture cardiac impulses. Anteroposterior chest and abdominal X-rays can localize the site where the pacemaker may have migrated. The dysfunctioning pacemaker must be replaced immediately after retrieval. Endovenous endocardial electrodes and subpectoral generator placement can be considered in older children.

Epicardial leads are often chosen for cardiac pacing in children either because of cardiac anatomy or because of the small size of the child. Advantages of epicardial leads are their easy applicability in every child, the possibility to combine the implantation of the leads with a corrective or palliative operation, fewer problems with the growth of the child, and the absence of the need for anticoagulation especially in children with a right-to-left shunt. Disadvantages of epicardial leads are the more extensive surgical procedure involved and the damage to the epicardial wall, which may result in difficulty in finding epicardium without scars for implantation of a subsequent epicardial lead. The epicardial leads also have shorter longevity compared with endocardial leads. Although endocardial pacing requires less extensive surgery than does epicardial lead implantation, there is concern about vascular obstruction, AV valve integrity, and the limitations of lead accommodation with somatic growth. Therefore a logical trade off would be to use steroid-eluting epicardial pacing leads especially in small children. Steroid leads, especially ventricular leads, significantly reduced battery drain and the potential for subsequent surgery.

Steroid-eluting epicardial pacing leads had a 2-year lead survival of 91 ± 5% (group I) as compared to 86 ± 7% (*P *= .97) with endocardial pacing leads in a study of 41 children who underwent pacemaker device implantation. The number of lead failures was 4 in both groups (*P *= .85). It was concluded that steroid-eluting epicardial leads have the same longevity as the conventional endocardial leads with similar pacing and sensing thresholds [7].

In another study based on two decades of experience, 71 patients (mean age, 5.3 ± 4.2, range: 1 day-16.2 years; mean body weight, 18 ± 12, range: 8–56 kg) underwent permanent pacemaker implantation of which 69% had epicardial pacing and in 31% of patients, transvenous pacing was established [8]. Age-adjusted Cox regression revealed no significant difference in reoperation rates among both groups. Battery depletion (n = 12) occurred after 3.4 ± 2.5 years. In 75% of patients, battery durability was less than four years. An increase in ventricular stimulation threshold resulting in the need for reoperation occurred in eight patients after 1.5 ± 1.8 years (2 days-4.5 years). In 75% of cases reoperation was necessary within 1.5 years after implantation. The age-adjusted rate of lead-related reoperation was significantly lower for patients with epicardial lead placement (P < 0.05). Lead-related reoperations were defined as reoperation due to early battery depletion (< 4 years), chronic stimulation threshold increase, lead fracture, lead dislocation, sensing dysfunction, and exit block [8].

Cohen and coworkers retrospectively reviewed 123 pediatric patients that underwent 207 epicardial lead insertion. They defined lead failure as the need for replacement or abandonment due to pacing or sensing problems, lead fracture, or phrenic and/or muscle stimulation. Epicardial lead failure occurred 16% of the time. The mean time to lead failure was 2.4 ± 2.3 years. The 1-, 2-, and 5-year lead survival was 96%, 90%, and 74%, respectively. Increasing threshold was the most common cause of lead failure. Only 2 (2.4%) steroid-eluting leads had to be abandoned for exit block. Compared with conventional epicardial leads, both atrial and ventricular steroid leads had better stimulation thresholds 1 month after implantation; however, only ventricular steroid leads had improved chronic pacing thresholds (at 2 years: for steroid leads, 1.9 μJ [from 0.26 to 16 μJ]; for nonsteroid leads, 4.7 μJ [from 0.6 to 25 μJ]; *P *< 0.01). Ventricular sensing was significantly better in steroid leads 1 month after lead implantation (at 2 years: for steroid leads, 8 mV [from 4 to 31 mV]; for nonsteroid leads, 4 mV [from 0.7 to 10 mV]; *P *< 0.01). [9].

The excessive pacing thresholds and high incidence of exit block with conventional epicardial leads presumably arise from a combination of epicardial fibrosis, scar formation, and/or pericardial adhesions after cardiac surgery. Conventional nonsteroid epicardial leads are associated with a 45% risk of exit block when implantation thresholds exceed 0.9 V at 0.5 ms. [9]. Cohen et al also confirmed the earlier observation that discharge excitation thresholds ≥ 3.0 μJ predicted lead failure [9]. To avoid this all pacing leads should be tested in the operating room.

Before the routine use of steroid leads, the 5-year epicardial lead survival was 40% to 70% [9]. In many series potentially pacemaker-related death is about 2% [10,11].

In the last ten years, 57 first time permanent pacemakers were implanted in the pediatric age groups at our institute. Steroid eluting epicardial leads were used in all at the first instance. Of these 57 cases, pacemaker migration occurred in the above three patients.

The three children in our series presented with varying degrees of intra-abdominal problems. The first child had intestinal obstruction due to entanglement of the pacemaker and its leads with the bowels. In the second child, electrical stimulation of the bowel, analogous to that of pectoralis major by axillary units, is theoretically possible, though mechanical irritation offers a more simple explanation. In the third child, the generator is likely to have eroded the colon gradually and then got extruded through the rectum. The process must have happened slowly, allowing time for sealing of the bowel wall and development of adhesions. The relatively large size of the pacemaker in a small malnourished child may have facilitated erosion into the bowel.

The complications reported here are rare but the risk of their occurrence would clearly be increased if the peritoneum and posterior rectus sheath were thin and fragile structures. Though not invariable, this situation may be present in children and in thin adult patients, particularly at the extremes of the age, who are also more likely to require abdominal units.

## Conclusion

We conclude that, whichever site for generator placement is chosen, such patients represent a special surgical problem which is only likely to be ameliorated by the development of progressively smaller generators in which prominent corners and lead attachments have as far as possible been eliminated. A disproportionately large size pacemaker is a major reason for migration especially in children. Appropriate fixation of the pacemaker in the pocket or preferably to an adjacent bony structure can help prevent migration. Despite all preventive measures, in cases when a pacemaker migrates, early detection can help avoid potential life-threatening complications. Lastly guidelines for permanent pacemaker implantation in children continue to evolve as developments in lead technology alter trends in pediatric cardiac pacing.

## Authors' contributions

HAS assisted the primary surgeon, conceived and designed the study, helped with literature review and manuscript preparation. RGM drafted the manuscript and the followed up the patients' perioperatively. MMM participated in the study design and preparation of the manuscript. JV was the primary cardiac surgeon who implanted the pacemaker devices, and was the overall supervisor and reviewer of the manuscript. All authors read and approved the final manuscript.

## Consent

Written informed consent was obtained from the respective parents regarding publication of the reports. A copy is available on request from the Editor-in-Chief.

## References

[B1] Cohen MI Rhodes LA Spray TL Gaynor JW Efficacy of prophylactic epicardial pacing leads in children and young adults Ann Thorac Surg 2004 78 197 202 10.1016/j.athoracsur.2004.02.008 15223427

[B2] Saha A Tan J Prendergast B Pacemaker lead fracture Heart 2003 89 783 10.1136/heart.89.7.783 12807857 PMC1767738

[B3] Abrams S Peart I Twiddler's syndrome in children: an unusual case of pacemaker failure Br Heart J 1995 73 190 2 Wong JK, House RJ. Pacemaker wire twist: Twiddler's syndrome. Australas Radiol 1993; 37:286–287 10.1136/hrt.73.2.190 7696033 PMC483790

[B4] Gomez C Dick M 2nd Hernandez R Coran AG Crowley D Serwer GA Peritoneal migration of an abdominally implanted epicardial pacemaker: a cause of intestinal obstruction Pacing Clin Electrophysiol 1995 18 2231 2232 10.1111/j.1540-8159.1995.tb04655.x 8771141

[B5] Dodge-Khatami A Backer CL Meuli M Prêtre R Tomaske M Mavroudis C Migration and colon perforation of intraperitoneal cardiac pacemaker systems Ann Thorac Surg 2007 83 6 2230 2 10.1016/j.athoracsur.2006.12.037 17532441

[B6] Koch AM Singer H Unusual pacemaker migration Eur Heart J 2005 26 1941 10.1093/eurheartj/ehi488 16169886

[B7] Beaufort-Krol GC Mulder H Nagelkerke D Waterbolk TW Bink-Boelkens MT Comparison of longevity, pacing, and sensing characteristics of steroid-eluting epicardial versus conventional endocardial pacing leads in children J Thorac Cardiovasc Surg 1999 117 523 8 10.1016/S0022-5223(99)70332-6 10047656

[B8] Sachweh JS Vazquez-Jimenez JF Schöndube FA Daebritz SH Dörge H Mühler EG Messmer BJ Twenty years experience with pediatric pacing: epicardial and transvenous stimulation Eur J Cardiothorac Surg 2000 17 455 61 10.1016/S1010-7940(00)00364-X 10773570

[B9] Cohen MI Bush DM Vetter VL Tanel RE Wieand TS Gaynor JW Rhodes LA Permanent epicardial pacing in pediatric patients: seventeen years of experience and 1200 outpatient visits Circulation 2001 103 2585 90 11382728 10.1161/01.cir.103.21.2585

[B10] Kerstjens-Fredrikse MWS Bink-Boelkens MTE de Jongste MJL Homan van der Heide JNH Permanent cardiac pacing in children: morbidity and efficacy of follow-up Int J Cardiol 1991 33 207 214 10.1016/0167-5273(91)90348-S 1743780

[B11] Esperer HD Singer H Riede FT Blum U Mahmoud FO Weniger J Permanent epicardial and transvenous single- and dual chamber pacing in children Thorac Cardiovasc Surg 1993 41 1 21 27 8367852 10.1055/s-2007-1013815

